# Isthmoceles — Accuracy of imaging diagnosis and clinical correlation with histology: A prospective cohort study

**DOI:** 10.52054/FVVO.16.2.021

**Published:** 2024-06-28

**Authors:** B Amro, M Ramirez, R Farhan, M Abdulrahim, Z Hakim, S Alsuwaidi, E Alzahmi, M Tahlak, P.R. Koninckx, A Wattiez

**Affiliations:** Latifa Hospital, Dubai 9115, United Arab Emirates; Dubai Hospital, Dubai, United Arab Emirates; Prof em OBGYN, Catholic University Leuven, Belgium, University of Oxford, Oxford, UK, Universita Cattolica del Sacro Cuore, Rome Italy; Department of OBGYN, Faculty of Medicine, University Strasbourg, 6081 Strasbourg, France

**Keywords:** Uterine Isthmocele, niche, caesarean scar defect, postmenstrual bleeding, MRI, transvaginal ultrasound

## Abstract

**Background:**

Isthmoceles are a growing clinical concern.

**Objectives:**

To evaluate the accuracy of diagnosis of isthmoceles by imaging and to correlate the dimensions with clinical symptoms and histopathology.

**Material and Methods:**

Prospective study of women (n=60) with ≥1 C-section undergoing hysterectomy. Isthmoceles were measured by imaging before surgery and macroscopically on the specimen after hysterectomy, followed by histological analysis.

**Main outcome measures:**

Accuracy of isthmocele diagnosis, correlation with clinical symptoms, and histopathological findings.

**Result:**

By imaging, isthmoceles were slightly deeper (P=0.0176) and shorter (P=0.0045) than macroscopic measurements. Differences were typically small (≤3mm). Defined as an indentation of ≥2 mm at site of C-section scar, imaging diagnosed 2 isthmoceles consequently not seen by histology and missed 3. Number of prior C-sections increased isthmocele severity but neither the incidence nor the remaining myometrial thickness (RMT) did. Severity correlated positively with symptoms and histology. However, clinical use was limited. Histological analysis revealed presence of thick wall vessels in 100%, elastosis in 40%, and adenomyosis in 38%. Isthmocele lining was asynchronous with the menstrual phase in 31%.

**Conclusions:**

Dimensions of isthmoceles by imaging were largely accurate with occasionally large differences observed. Number of C-sections did not increase isthmocele incidence, only severity. Indication for surgery remains clinical, considering dimensions and symptoms.

**What is new?:**

Dimensions of isthmoceles should be confirmed before surgery since uterine contractions might change those dimensions. Symptoms increase with dimensions of isthmoceles but are not specific. Endometrial lining within the isthmocele can be asynchronous with the menstrual phase.

## Introduction

Depression in the anterior uterine wall at the site of a previous caesarean section (CS) was described by Poidevin (1961) through hysterosalpingography and by Morris (1995) through pathology. The terms used varied between isthmocele, niche, caesarean scar defect, and caesarean scar dehiscence (Wang et al., 2009; Bij de Vaate et al., 2010; Vikhareva Osser and Valentin, 2010; Bij de Vaate et al., 2011; van der Voet et al., 2014a). Clinical interest has increased exponentially, with 24.210 publications in PubMed in the last five years (accessed September 12, 2023), and several excellent recent reviews (Donnez, 2023; Klein Meuleman et al., 2023; McGrattan et al., 2023; Spong et al., 2023; Verberkt et al., 2023). The diagnosis is made by ultrasonography with or without uterine distention, magnetic resonance imaging (MRI), hysteroscopy, or direct macroscopic measurements on hysterectomy specimens (Bij de Vaate et al., 2011; Tulandi and Cohen, 2016; van der Voet et al., 2017; Jordans et al., 2019b; Vikhareva et al., 2019). To standardise the definition, a European panel suggested in 2019 the definition of “an indentation at the site of the caesarean section scar with a depth of at least 2 mm at ultrasonographic evaluation” (Jordans et al., 2019a). Emphasising that isthmocele is a clinical entity, a panel of 31 international experts agreed on a consensus that symptoms should be added to the definition. Therefore, they introduced the term caesarean scar disorder (CSD), which is defined as a uterine niche in combination with at least one primary or two secondary symptoms (Klein Meuleman et al., 2023). Unfortunately, it remains unclear which isthmoceles are clinically significant and what is the optimal treatment (Mc Gowan et al., 2022; Armstrong et al., 2023; McGrattan et al., 2023).

The pathophysiology of isthmoceles is unclear (Vervoort et al., 2015; de Luget et al., 2022; Stegwee et al., 2023). It is speculated that they result from improper healing caused by surgical technique, the body’s healing mechanisms, or C-section-related complications. The incidence after a C-section was reported to increase with smoking, advanced gestational age, twin pregnancy, double-layer closure, and less experienced surgeons. Previous vaginal delivery and the use of Vicryl sutures have been shown to be protective (Stegwee et al., 2023). Postpartum alpha-lipoic acid supplements were suggested to improve uterine scar healing and reduce the incidence of isthmocele (Sammour et al., 2019). It is unclear whether double-layer closure of uterine incision decreases the incidence of isthmocele (Roberge et al., 1999; de Luget et al., 2022), with some studies reporting a lower incidence (Genovese et al., 2023) while others not finding a difference (Stegwee et al., 2020; Stegwee et al., 2023). To date, it remains unclear how isthmoceles can be predicted or prevented.

The reported incidences of isthmoceles vary widely and depend on the method of diagnosis and population studied. The incidence varies between 19% and 84% by ultrasound examination (van der Voet et al., 2014b). The reported incidence is 71% in the Netherlands (Stegwee et al., 2023) and 45% in China (Pan et al., 2019). With sonohysterography, the variability of reported incidence is lower between (56% to 78%), and by hysteroscopy it is 75% (van der Voet et al., 2017). The severity increases with the number of C-sections (Vikhareva Osser et al., 2009; Wang et al., 2009), whether assessed at 2 months or one year after a C-section (van der Voet et al., 2018). The apparent increase over time in isthmocele incidence might reflect the increase in caesarean section rates worldwide and the increasing interest and awareness among gynaecologists and radiologists (Barber et al., 2011; Vervoort et al., 2015).

Clinical symptoms associated with isthmoceles vary widely, and our understanding remains poor. Symptoms have been shown to relate to isthmocele width (Wang et al., 2009). Frequent symptoms such as irregular bleeding and postmenstrual bleeding have been explained as retention of menstrual blood within the isthmocele or local bleeding from new and fragile blood vessels (Tulandi and Cohen, 2016). Dysmenorrhea, dyspareunia, and chronic pelvic pain were suggested to be caused by fibrosis and impaired drainage during menstruation (Wang et al., 2009; van der Voet et al., 2014a; Vikhareva et al., 2019). Infertility might be caused by impaired sperm function or motility due to ‘hostile ’fluid in the isthmocele (Bij de Vaate et al., 2011; Gubbini et al., 2011; Tulandi and Cohen, 2016). Caesarean scar pregnancy could be both a cause or a consequence of a large isthmocele (Jordans et al., 2022). Unfortunately, uterine scar rupture during pregnancy remains unpredictable (Tanos and Toney, 2019).

The histology of the isthmoceles has received relatively little attention. To date, only 56 hysterectomy specimens (Morris, 1995, Karpathiou et al., 2020) in the medical literature have been examined for isthmoceles. All other examinations were performed on resected specimens during hysteroscopy or laparoscopy (Gubbini et al., 2011; Donnez et al., 2017; Shapira et al., 2020; Zhu et al., 2020; Abdullgaffar and Almulla, 2022; Higuchi et al., 2021). The correlation of histology with clinical symptoms has not yet performed.

Despite the interest and many reviews, it remains unclear which symptoms or dimensions of isthmoceles are an indication for surgery. In addition, the accuracy of the diagnosis by imaging, is not well established since it has not been directly compared to other accurate method. Therefore, we estimated the accuracy of imaging measurements of isthmoceles by comparing them with direct macroscopic measurements after hysterectomy. As a secondary aim, we analysed the correlation between those dimensions with the severity of symptoms and their clinical impact.

## Materials and methods

### Study design and population

The primary aim was to evaluate the accuracy of the diagnosis of isthmocele by imaging. Therefore, isthmoceles were measured by imaging (ultrasound or MRI) before surgery and macroscopically on the uterine specimen after hysterectomy. The secondary aim was to evaluate how clinical symptoms of isthmoceles correlated with histopathological findings and dimensions. Included were all women (N=60) with a history of one or more C-sections undergoing a laparoscopic hysterectomy at Latifa Hospital, Dubai, between October 2019 and September 2022. Indications for hysterectomy were menorrhagia or abnormal uterine bleeding, adenomyosis, myoma, chronic pelvic pain, endometrial hyperplasia, or a combination of these. The presence of an isthmocele alone was not considered an indication for hysterectomy. Excluded were women with uterine cancer, or when morcellation for uterine extraction was anticipated since this would prevent proper macroscopic measurements and histological analysis.

**Figure 1:**
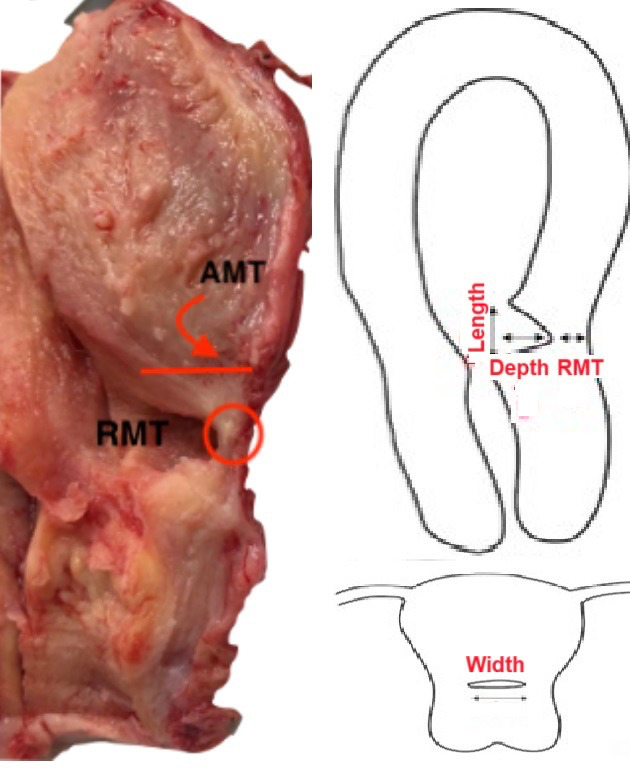
Definitions of depth, length, width, residual myometrial thickness (RMT) and adjacent myometrial thickness (AMT) of an isthmocele. Width needs to be measured in a transverse plane, which can be difficult by transvaginal ultrasound.

Before surgery, the severity of clinical symptoms such as postmenstrual bleeding, postmenstrual discharge, dyspareunia, and dysmenorrhea were recorded on a 0-3 scale (Biberoglu and Behrman, 1981). Also, women’s age, parity, number of previous C-sections, and secondary infertility (Y/N) were recorded. Isthmoceles were evaluated by imaging (US or MRI). Their shape was described, and the length (L), depth (D) and residual myometrial thickness (RMT) were measured. The length equates to the widest gap along the longitudinal axis, the depth with the vertical distance between the defect’s base and apex and the residual myometrial thickness with the distance between the apex of the defect and the outer myometrium at the sagittal plane (Figure 1).

This prospective study was approved by the Dubai Scientific Research Ethics Committee (DSREC) of Dubai Health Authority (DHA), Dubai, UAE, as DSREC-10/2019_23. Informed consent was obtained before inclusion. Results are reported according to the STROBE checklist.

### Histopathology analysis

The 10% formalin-fixed uterine specimens were weighed. The length, width and thickness of anterior and posterior uterine walls were measured. Myomas, adenomyosis, polyps, and endocervical inclusion cysts were documented. For isthmoceles, the length (L), depth (D), width (W), which were taken at the transverse plane, and residual myometrial thickness (RMT) were measured. In addition, the location (isthmus or upper/lower cervical canal), shape (semicircular, triangular, or tubular), and myometrial thickness adjacent to isthmocele (AMT) were documented (Figure 1). These dimensions permitted volume calculation using (L*W*D)/2 for triangular isthmoceles and (π*D2*W)/2 for semicircular isthmoceles. Isthmoceles were serially sectioned transversely at 2 mm intervals and stained with Hematoxylin and Eosin (H&E). Masson Trichrome Histochemical and Actin Immunohistochemical stains were used if needed. Histological findings were documented such as thick-walled vessels, congested vessels, haemorrhage, type of epithelial lining, foreign body giant cell reaction, inflammatory cells, lymphocytic infiltration, adenomyosis and elastosis (which is the focal globular accumulation of the thick elastic fibres). The endometrial lining in the isthmocele as well as the rest of the uterine cavity was evaluated histologically and hence labelled as proliferative phase, secretory phase, or atrophic.

### Statistical analysis

The first and second questionnaires were answered Statistical analysis was done with SAS statistical software (SAS, 2020) using descriptive statistics, and Chi-square, correlations (Spearman), paired t-test, and Wilcoxon as appropriate. According to the recommendations of the American Statistical Association (Wasserstein and Lazar, 2016), exact P-values and, unless indicated otherwise, medians (10th – 90th percentiles) are given.

## Results

### General data

A total of 60 eligible women accepted to participate in this study spanning over three years. Their demographics are listed in Table I. Median age was 48 (range, 41-58) years. 33%, 25%, 18% and 23% had undergone one, two, three, or ≥4 C-sections, respectively. In our series, last caesarean section had been performed 12 (range, 5-24) years previously.

**Table I:** Clinical characteristics of the study population.

Number of patients	60*
Age, year	48 (41-58)
Parity	9.5 (1-12)
No. of previous cesarean section	
1	20
2	15
3	11
≥ 4	14
Time since last CS (years)	12 (5-24)
Retroversion of uterus	21
Clinical symptoms	
Dysmenorrhea	35 ≥2/3 score
Dyspareunia	34 ≥2/3 score
Postmenstrual spotting	33 ≥2/3 score
Chronic vaginal discharge	22 ≥2/3 score
Secondary infertility	10

*Values are given as median (10th-90th perentile) or as number of patients.

**Figure 2:**
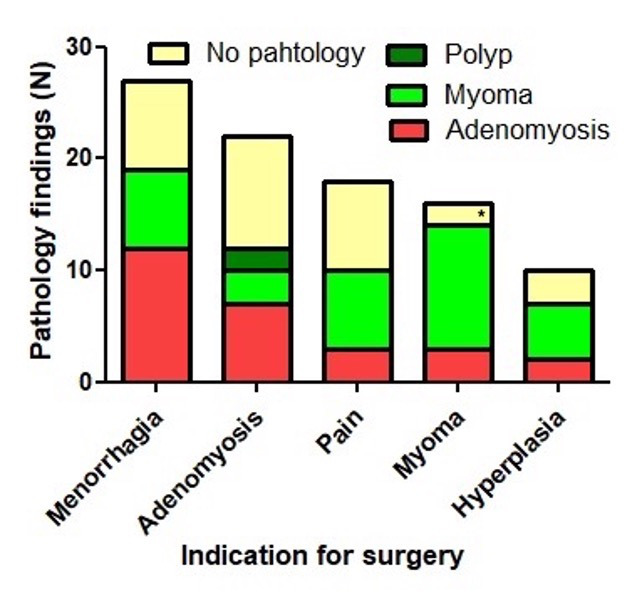
Indications for hysterectomy and findings by pathology. The primary indications for hysterectomy were menorrhagia, adenomyosis, pain, myoma, or endometrial hyperplasia. The main pathology findings (polyp, myoma, adenomyosis, no clear pathology) were shown by colours and plotted against each column. Both were correlated poorly. For example, when the primary indication for hysterectomy was adenomyosis, the histological finding was adenomyosis in 27% (red colour). These data illustrate that the indication for hysterectomy and the pathology are essentially multivariate since most women have multiple symptoms and histological observations. The presence of isthmoceles could be another variable. * One case had been diagnosed as large subserous myoma preoperatively (by US), but the histology showed no pathology in the uterus. It was an ovarian fibroma.

The hysterectomy’s primary indications were menorrhagia and abnormal uterine bleeding (AUB) in 40%, adenomyosis in 22% and myoma in 17%. The correlation between the indication and histological findings was weak (Figure 2). For example, when the primary preoperative diagnosis was adenomyosis, the histological finding was adenomyosis in just 27% and polyps, myoma or no clear pathology in 15%, 8%, and 50%, respectively. The presence of an isthmocele may have contributed to the symptoms before the hysterectomy. However, isthmoceles alone were not considered an indication for hysterectomy. The clinical symptoms were not specific and often occurred simultaneously: dysmenorrhea ≥2/3 in 58%, dyspareunia in 56%, postmenstrual bleeding sin 55%, chronic vaginal discharge in 37%, and secondary infertility in 17%. The overlapping of symptoms emphasises the difficulty of analysing the symptoms of an isthmocele since it requires a multivariate analysis, which cannot be applied at our sample size.

### Isthmocele measurements by imaging (US and MRI) and macroscopically by histology

**Figure 3:**
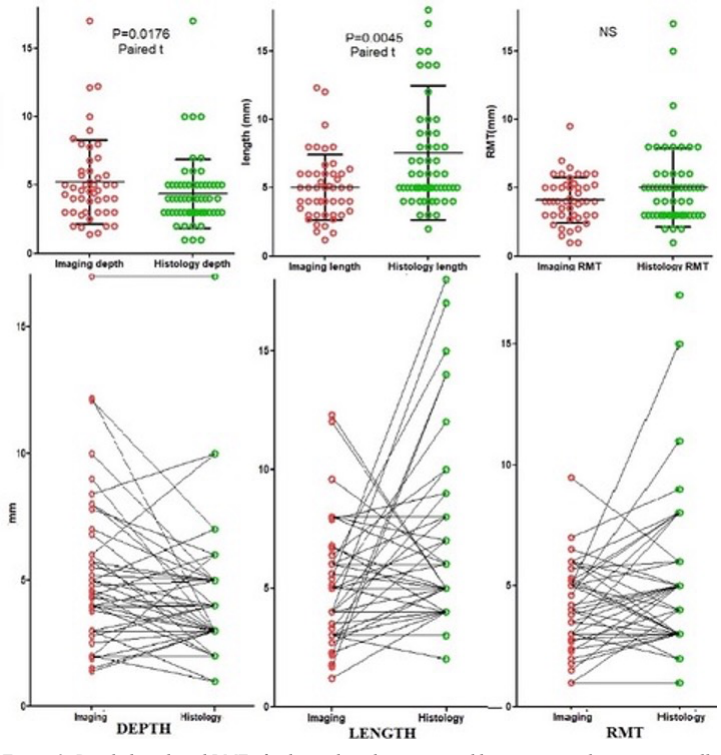
Depth, length and RMT of isthmoceles when measured by imaging and macroscopically on uterine specimen. The upper graphs show the Mean + 1 SD and the individual measurements by imaging (red color) and histology (green color); isthmoceles were deeper (P=0.018) and shorter (P=0.0045) by imaging compared to its histology (macroscopic) measurements (Paired t-test). The lower graphs illustrate how those measurements correlate individually with each other by imaging and histology. This correlation is strong for depth (P=0.034) and RMT (P=0.0084) but not for length. It also demonstrates the small variations in those individual measurements around 1-3mm. However, in some women, differences can be up to 10 mm. Therefore, it is suggested to repeat imaging before deciding on surgery.

**Figure 4:**
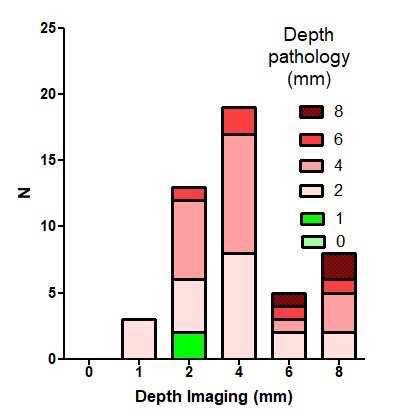
Diagnosis of isthmocele based on depth measurements by imaging and pathology (macroscopically): Imaging measurements (mm) illustrated with a frequency distribution (bars) and in each of those bars, the pathology measurements (mm) illustrated by different colors. Using the isthmocele definition as depth ≥ 2mm; imaging missed 3 isthmoceles (first column from left that showed 1mm by imaging while the color is pink (2mm by pathology)) and diagnosed 2 isthmoceles (second column from left that showed 2mm by imaging with green color representing 1mm by pathology). This also explains that isthmocele prevalence at our series was 94% by imaging and 95% by pathology (macroscopically). Equally important is to realise that deep isthmoceles of 8mm by imaging (last column from right) vary between 2 and 8 mm by pathology (represented by colors at that column). Suggesting repeat imaging measurements for confirmation before deciding on surgery.

**Figure 5:**
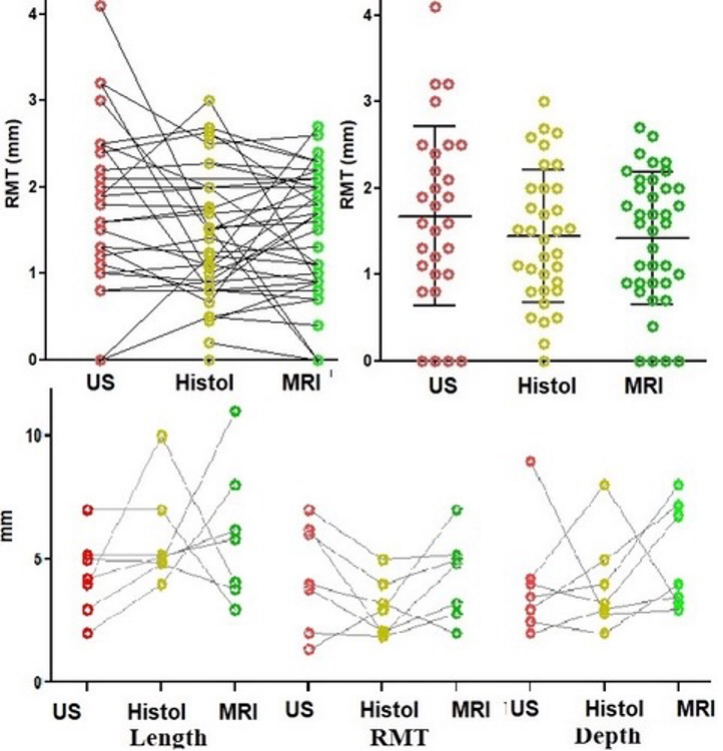
Comparing Isthmocele measurements by US, MRI, and histology. This was done for the 7 cases who had both US and MRI pre-hysterectomy (lower graph). It showed the frequent differences of a few mm between imaging and macroscopic measurements and between US and MRI. In the upper graph we integrated the data of Donnez et al. (2017) for RMT. It seems that RMT measurements by MRI are more accurate when compared to US, although not statistically significant, as seen in the upper right graph.

We aimed to standardise imaging before hysterectomy with utilising both US and MRI for each case. Unfortunately, this was not possible due to insurance providers refusing coverage and extra costs to patients. Therefore, imaging was classified as Transvaginal Ultrasound alone (n=38), MRI alone (n=15) and both modalities (n=7). Considering the small numbers for each type of imaging, we combined them both for the initial analysis. By imaging, isthmoceles were deeper (P=0.017) and shorter (P=0.0045) than when measured macroscopically. In individual women, most measurements differed only by 1-3 mm and occasionally by more than 5-10 mm (Figure 3). Applying the criteria of depth ≥ 2mm for diagnosis of an isthmocele, these differences affect the prevalence of isthmocele being 94% by imaging and 95% macroscopically by histology. Imaging did not diagnose 3 isthmoceles which were diagnosed histologically; and on the other hand, imaging also diagnosed 2 isthmoceles which were not also diagnosed macroscopically (Figure 4). We compared the measurements in the 7 cases who underwent both US and MRI to their respective macroscopic measurements. RMT measurement by MRI was slightly more accurate than US when compared to the histological data, although this was not a statistically significant difference (Figure 5).

### Incidence, location, shape, and dimensions of isthmocele

**Figure 6:**
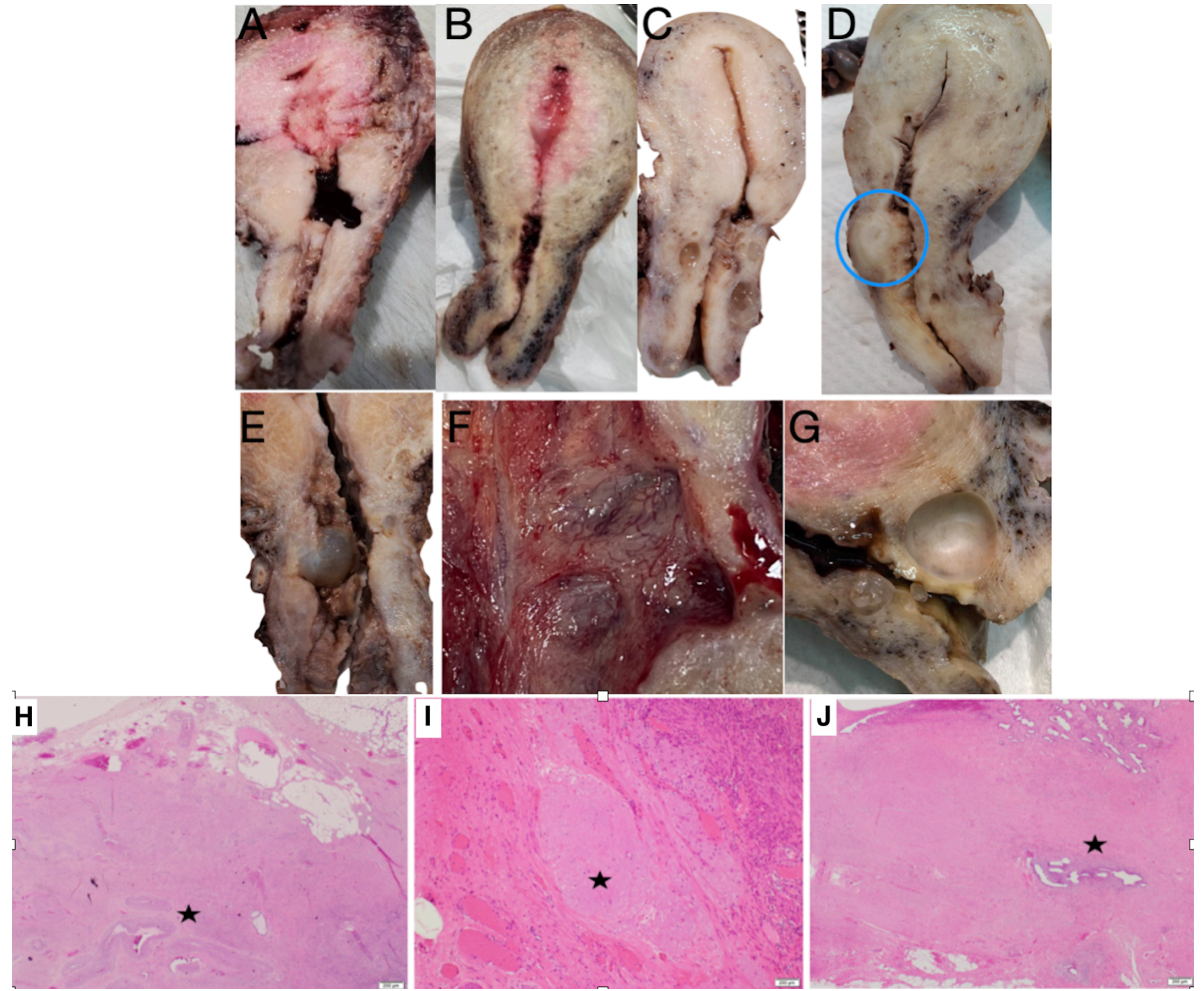
Histology of isthmocele; macroscopic (upper image) and microscopic (lower image) illustration. Upper image: (A) tubular, (B) semicircular, and (C) triangular isthmoceles. (D) Rare finding of myoma at isthmocele body. (E) Inclusion cyst at isthmocele. (F) Prominent neovascularisation at isthmocele lining. (G) large Nabothian close to the isthmocele. Lower image: (H) thick-walled vessels (HE, 20X). (I) elastosis; pale area of degenerative changes (HE, 100X). (J) Adenomyosis (HE, 20X). All at the residual myometrium of isthmocele (RMT).

**Figure 7:**
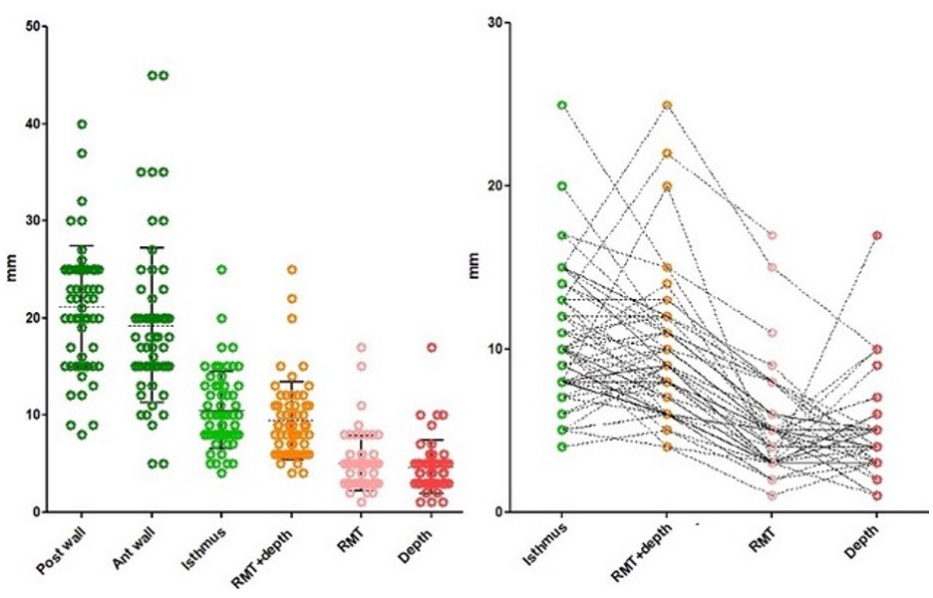
Histology (macroscopic) measurements of anterior uterine wall thickness (18 mm,10.5-30), posterior uterine wall thickness (22 mm, 13.5-26.5) and isthmus or adjacent myometrial thickness (AMT) (10 mm, 6-15). The sum of RMT and depth of the isthmocele (RMT+ depth) is similar to the AMT (Isthmus), thus indirectly confirming the accuracy of those measurements. The right graph shows the individual measurements in relation to each other. Occasional variations can be noticed. It also showed that deeper isthmoceles (red dots) are associated with thinner RMTs (pink dots).

The number of C-sections did not increase the incidence of isthmoceles, being 100%, 82% and 95% by imaging and 66%, 100% and 33% by pathology after 1, 2 or ≥3 C-sections, respectively. However, the number of C-sections increased the width (P=0.042) and decreased the ‘healing ratio’ (residual myometrial thickness (RMT)/adjacent myometrial thickness (AMT) - (P=0.045), but not the residual myometrial thickness (RMT) per se. Isthmoceles were located at the upper or lower cervical canal in 85% and 15% of the cases, respectively. Most isthmoceles were triangular (81%), sometimes semicircular (17%), and rarely tubular (2%) (Figure 6). Semicircular isthmoceles had larger volumes (713 + 309 ml) than triangular ones (243 + 80 ml). Two women had two isthmoceles, and both were semicircular. A retroverted uterus, found in 35% of our cases, did not correlate with the number of CS or isthmocele shape, dimension, and histology. Isthmocele width by macroscopic measurement was the common factor impacting all other measurements such as length, depth, and volume, i.e., wider isthmoceles were longer (P=0.0025), deeper (P=0.027) and larger (P<0.0001). Deeper isthmoceles associated with thinner RMT (Figure 7).

### Symptoms

By univariate analysis, the shape or dimension of isthmoceles did not predict symptoms, which is not surprising considering the other uterine pathologies in these women. Since a multivariate analysis cannot be performed in 60 women, only observations are listed. Bleeding and chronic vaginal discharge were associated with each other (P=0.0007). Dyspareunia was associated with dysmenorrhea (P=0.015). Retroversion of the uterus did not increase the isthmocele severity or dimensions, although was associated with dyspareunia (P=0.025) and, to a lesser extent, dysmenorrhea (P=0.039). Longer isthmoceles were associated with dyspareunia (P=0.048) and a reduced incidence of secondary infertility (P=0.014). Shallow isthmoceles were associated with dysmenorrhea (P=0.020).

### Histology

The histological examination of the isthmocele lining did not clearly correlate with symptoms and provided no additional clinical value. However, the histological observations may provide better understanding of the underlying pathophysiology. Frequent findings included congestion of capillaries in 99% and free haemorrhage in 83%, and both showed a positive correlation with each other (P<0.0001). These histological findings were associated with dysmenorrhea (P=0.0035 and P= 0.020), dyspareunia (non-significant and P=0.020), the years since the last C-section (P=0.008 and P=0.043), and with less chronic vaginal discharge (non-significant and P=0.019). Chronic lymphocytic infiltration, found in 55%, was only associated with uterine weight (P= 0.0025). Wider isthmoceles were associated with foreign body reaction (P=0.024), chronic vaginal discharge (P=0.0009), and adhesions at the caesarean scar site (P=0.004), but with less bleeding (P=0.037). The isthmocele lining was endometrial mucosa in 70%, mixed endometrial and endocervical mucosa in 15%, and atrophic in 15%. Elastosis, a focal globular accumulation of thick elastic fibres suggestive of an altered wound-healing process, was found in 38% and was associated with longer (P=0.044) and deeper (P=0.043) isthmoceles. Its incidence decreased with the number of C-sections, being 75%, 41% and 37% after 1,2, ≥ 3 C-sections respectively (Manzel-Haenzel P<0.001). Elastosis also increased with the duration of time since the last C-section (P=0.043). Thick-walled vessels at the isthmocele body were very prominent in 78% and associated with infertility (P=0.03). Adenomyosis, defined by isolated endometrial mucosa and glands in the residual myometrium of the isthmocele, was found in 38% (Figure 6) but did not correlate with symptoms or adenomyosis in the uterine myometrium.

Interestingly, in our series, we did not find a real hanging endometrial fold at the edge of the isthmocele and we observed that the endometrium in the isthmocele was asynchronous with the remainder of uterine endometrium in 31%. A proliferative uterine endometrium was associated with a proliferative, secretory, and atrophic isthmocele lining in 82%, 8% and 10%, respectively; while a secretory uterine endometrium was associated with a proliferative, secretory, and atrophic isthmocele lining in 65%, 20% and 15%, respectively.

## Discussion

Recently the definition of an isthmocele has varied from an indentation ≥2mm to clinical symptoms together with this indentation (Jordans et al., 2022; Klein Meuleman et al., 2023). Unfortunately, both definitions resulted from experts’ opinions without validation. We confirmed that symptoms are not specific to isthmoceles and overlap with several uterine pathologies, that symptoms poorly correlate with isthmocele depth, and that often several symptoms are simultaneously present. It is unlikely that a depth of isthmocele or a set of symptoms with clinical diagnostic value can be defined. Although the histopathology of isthmocele lining might predict the effectiveness of hysteroscopic resection (Zhu et al., 2020), it has little additional clinical value. Pathology is necessary to exclude malignancy or a polyp-like disorganised local mucosa (Karpathiou et al., 2020). However, we found only one small polyp in this series. A multivariate analysis might be helpful to understand the mechanisms involved but cannot be expected to add much to diagnostic accuracy. Therefore, since it is unclear which depth of indentation constitutes pathology, the indication for surgery remains a clinical decision, considering the dimensions of an isthmocele as well as the severity and type of symptoms.

The incidence of isthmoceles thus varies with the definitions and the diagnostic methods used. Our data confirm that the incidence does not increase with the number of C-sections, although severity does, as observed previously (Wang et al., 2009).

The dimensions such as depth, length, width, and RMT are important for understanding isthmoceles, clinical decision-making, and possibly prevention. Therefore, the accuracy of these estimations is essential. Imaging is the gold standard since it is a non-invasive method. However, the accuracy is unclear if not validated in comparison with a more accurate method, such as direct measurement of uterine specimens following hysterectomy. Our data confirm the accuracy of imaging measurements to within 1 to 3 mm in most women. However, depth by imaging can occasionally be exaggerated (≥5 mm). We suggest, although speculatively, that uterine contractions might cause those exaggerated depths while acting at the weakest point, i.e., the isthmocele. Therefore, we suggest repeating the isthmocele measurements before deciding for surgery. This is even more important for smaller isthmoceles since with its definition of an indentation ≥2mm, an error of 1 or 2 mm changes the diagnosis. It is suggested to be cautious here to avoid exposing the woman with previous C-sections and minor symptoms to possible unnecessary procedures such as hysteroscopy or laparoscopy.

The pathophysiology of isthmoceles remains unclear. Retroversion of the uterus was not found to be associated with deeper isthmoceles, although suggested previously (Vikhareva Osser et al., 2009; Wang et al., 2009; Vikhareva Osser and Valentin, 2010; Bij De Vaate et al., 2011; Roeder et al., 2012; Bij De Vaate et al., 2014). It was speculated that contractions of the archimetra (Leyendecker et al., 2023) during healing play a role since wound healing can be abnormal in moving tissues, leading to fibrosis (Roeder et al., 2012; Cramer and Heller, 2018). This hypothesis seems consistent with our observation that elastosis is associated with longer and deeper isthmoceles. Impaired myometrial contractility because of fibrotic tissue and microtears with possible involvement of nerve endings at the myometrium (Roeder et al., 2012; Cramer and Heller, 2018), could explain the associated pain.

Although speculative, uterine contractility might be the missing link in the pathophysiology of isthmoceles and the associated pain and bleeding. Smaller isthmoceles might reflect only scar retraction without being pathological. We confirmed the frequent presence of haemorrhage, congestion, and lymphocytic infiltration (Morris, 1995; Gubbini et al., 2011; Donnez et al., 2017; Shapira et al., 2020; Karpathiou et al., 2020; Abdullgaffar and Almulla, 2022). Foreign body giant cell reaction was observed in 22%, although previously described in 92% (Morris, 1995) which may reflect improvement in suture materials. Elastosis decreased with number of C-sections and increased with time since the last C-section, pointing to the prolonged wound healing process and remodelling of a C-section scar. Elastosis was not confined to the first 1-2 years after C-section, as suggested before (Roeder et al., 2012).

We observed adenomyosis in the RMT in 38% of the cases, which is comparable to the 28% reported after hysterectomy (Morris, 1995), to 21% in laparoscopically-resected specimens (Donnez et al., 2017) and 12% in hysteroscopically- resected specimens (Shapira et al., 2020). This adenomyosis is probably iatrogenic rather than a ‘true’ adenomyosis as it was not associated with the presence of adenomyosis at the rest of the uterine myometrium. This is consistent with the absence of a statistically significant association between adenomyosis in RMT and clinical symptoms such as pain in our study. This was a debatable issue since histology in those studies was for the resected isthmoceles and not the whole uterus (Shapira et al., 2020; Donnez et al., 2017).

What is new in our series is that we did not find the common phrase of “overhanging endometrial folds” at isthmocele edges as described previously in 61% of cases (Morris, 1995). This is important since overhanging endometrial folds are used as an argument to resect the isthmocele borders by hysteroscopy (Gubbini et al., 2011; Raimondo et al., 2015; Shapira et al., 2020). Another important new observation is that the RMT did not vary with the number of C-sections, thus questioning the prevailing concept that multiple C-sections increase the risk of scar rupture due to thinner RMT. This supports the importance of avoiding prophylactic correction of isthmocele in asymptomatic women just to increase the RMT to decrease the risk of scar rupture in future pregnancy. A remarkable and new observation also is the 31% discrepancy between the endometrium lining the isthmocele and the uterine cavity. Atrophy might be explained by poor vascularisation. The absence of secretory changes could be explained by progesterone resistance, or basal endometrium (Koninckx et al., 2022). It is challenging to explain the asynchronous secretory changes found in the isthmocele compared to the endometrial phase at uterine cavity in the absence of progesterone. Therefore, it is tempting to consider a different tissue reaction that explains the poor response of isthmocele-related symptoms to systemic hormonal treatment.

In conclusion, most women have an isthmocele after a C-section, and the severity hardly increases with the number of C-sections. The 2 to 3 mm differences, or more, between imaging and direct measurements, advocate for repeat imaging before deciding for surgery. For smaller isthmoceles, this can prevent the vicious circle of surgery for minor symptoms in those women. The pathophysiology of isthmoceles remains poorly understood. However, we speculatively posit that uterine contractions might be the missing link. We question whether edge resection is indicated during hysteroscopic treatment of isthmocele when no overhanging endometrial folds are seen. However, base resection may be of more value considering the many abnormalities seen in that area, such as thick wall vessels and elastosis. Prophylactic correction of asymptomatic thin-walled isthmoceles (RMT) by imaging to decrease the risk of scar rupture in future pregnancies is questionable.
